# 

**Published:** 1860-09

**Authors:** 


New Series, Vol. 3, No. 9. Whole Series, Vol. 17, No. 9.

THE CHICAGO

### MEDICAL JOURNAL.

DANIEL BRAINARD, M. D.,

EPHRAIM INGALS, M. D.,

EDITORS AND PROPRIETORS.

SEPTEMBER, 1860.

CHICAGO:

J. W. HYATT, Jr., BOOK AND JOB PRINTER, 23 LAKE STREET,

To whom all advertisements for insertion in the Journal may be addressed.

PUBLISHED MONTHLY, AT TWO DOLLARS PER ANNUM, IN ADVANCE.

